# Non-reciprocal spin excitations across the skyrmion–paramagnetic phase transition in MnSi

**DOI:** 10.1107/S1600576725011537

**Published:** 2026-02-17

**Authors:** T. Weber, K. Schmalzl, J. Waizner, A. Bauer, M. Garst, C. Pfleiderer

**Affiliations:** a Institut Laue–Langevin (ILL), 71 avenue des Martyrs, 38000 Grenoble, France; b Jülich Centre for Neutron Science (JCNS), Outstation at ILL, 71 avenue des Martyrs, 38000 Grenoble, France; c Institut für Theoretische Physik, Universität zu Köln, Zülpicher Straße 77a, 50937 Köln, Germany; d Physik-Department Technische Universität München (TUM) James-Franck-Straße 1 85748 Garching Germany; e Karlsruhe Institute of Technology (KIT), 76131 Karlsruhe, Germany; f Forschungsneutronenquelle Heinz-Maier-Leibnitz (MLZ), Lichtenbergstraße 1, 85747 Garching, Germany; g Munich Center for Quantum Science and Technology (MCQST), Schellingstraße 4, 80799 Munich, Germany; h Zentrum für QuantumEngineering (ZQE), Am Coulombwall 3a, 85748 Garching, Germany; Technical University of Denmark, Denmark

**Keywords:** magnons, skyrmions, inelastic neutron scattering, linear spin-wave theory, MnSi, skyrmion–paramagnetic phase transitions, non-reciprocal

## Abstract

The magnetic excitations of the skyrmion lattice in MnSi comprise a multitude of individual modes, which are non-reciprocal and thereby propagate unidirectionally. We report inelastic neutron scattering experiments for temperatures near and above the skyrmion–paramagnetic phase transition in the chiral magnet MnSi, tracking the evolution from the skyrmion lattice towards the high-temperature paramagnetic state. Within the resolution of the triple-axis measurements the excitations vary smoothly across the skyrmion–paramagnetic boundary, and the quasi-elastic paramagnetic signal under applied field retains the non-reciprocal character seen in the skyrmion phase even far above the critical temperature. Using a resolution convolution, our results are consistent with linear spin-wave theory.

## Abstract 2

Inelastic neutron scattering shows that the non-reciprocal nature of the magnons in the skyrmion phase of MnSi is maintained for high temperatures well beyond the skyrmion–paramagnetic phase transition.

## 1. Introduction

Below temperatures of *T*_c_ ≈ 29 K, the itinerant-electron compound MnSi features several magnetically ordered phases (Bauer & Pfleiderer, 2012 ▸; Bauer *et al.*, 2013 ▸), which have drawn great interest in the past decade. The magnetic phase diagram consists of helical, conical and field-polarized ferromagnetic states, as well as a skyrmion phase that is topologically distinct in terms of its non-zero winding number (Mühlbauer *et al.*, 2009 ▸). In the helical phase below a critical field 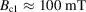, four helical domains align along the [111] easy axes (Ishikawa *et al.*, 1976 ▸). Increasing the field above *B*_c1_ aligns the multiple domains into one single domain, with the helix propagation vector ordering along the applied field direction (Mühlbauer *et al.*, 2009 ▸). Further increasing the field causes a conical canting of the spins towards the field direction until, above the second critical field 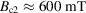, they are fully aligned with the external field.

Spontaneous long-range order vanishes above a transition temperature of *T*_c_ ≈ 29 K (Ishikawa *et al.*, 1985 ▸; Roessli *et al.*, 2002 ▸). A fluctuation-disordered paramagnetic regime exists between the long-range ordered phases and the paramagnetic regime at high temperatures, in which the discrete magnetic satellites of the ordered phases spread out evenly over the surface of a sphere centred around the nuclear Bragg reflections (Janoschek *et al.*, 2013 ▸). It is disputed whether the fluctuation-disordered phase serves as a precursor of the ordered phases (Pappas *et al.*, 2017 ▸). A phase of twisted magnetic spirals similar to the blue phase of liquid crystals has been reported to persist beyond *T*_c_ (Hamann *et al.*, 2011 ▸).

Studies of the spin excitations of the ordered phases of MnSi go back to the 1970s with the pioneering work of the Ishikawa group (Ishikawa *et al.*, 1977 ▸). They could decipher the general parabolic form of the dispersion, but their experiments were not sensitive to the details of the dispersion branches. In later high-resolution measurements, magnetic excitations in the helical phase were found to take the form of a band structure for momentum transfers **q** perpendicular to the helix propagation vector and of two non-reciprocal, as well as one central, symmetric modes for momenta along the helix (Janoschek *et al.*, 2010*a* ▸; Kugler *et al.*, 2015 ▸; Weber *et al.*, 2018*b* ▸, 2019 ▸). The magnon band structure of the helical and conical phases originates from a strong back-folding of the spectra into the first magnetic Brillouin zone (Garst *et al.*, 2017 ▸), which – for MnSi – is ∼35 times smaller than the first nuclear zone.

Non-reciprocity in the simplest scenario alludes to magnon dispersion, where magnons are created at different magnitudes of energy than they are annihilated at (Sato & Matan, 2019 ▸). It may be the consequence of a non-centrosymmetric crystal structure – MnSi crystallizes in the *P*2_1_3 space group – and the ensuing Dzyaloshinskii–Moriya interaction together with the broken time-reversal symmetry due to a spin order that is, in the present case, imposed by an external magnetic field (Sato & Matan, 2019 ▸). The investigation of non-reciprocal magnon dispersions holds great potential in the research of magnonic devices where the propagation of magnons in one direction is required, such as spin-wave diodes (Szulc *et al.*, 2020 ▸) or directional couplers (Tian *et al.*, 2025 ▸).

The asymmetry of the non-reciprocal modes in MnSi becomes more pronounced in the conical phase, with the spectral weight shifting from one of the modes centred on a magnetic satellite to another, until only a single mode remains in the fully field-polarized phase, This phenomenon was first mentioned in the 1980s (Shirane *et al.*, 1983 ▸). Interestingly, the mode in the field-polarized ferromagnetic state rests centred on a position in momentum space where one of the magnetic satellites of the conical phase would be, even though the field-polarized phase is commensurate and thus no satellite peaks remain (Weber *et al.*, 2018*b* ▸).

First investigations into the excitations of the paramagnetic phase were conducted in the 1980s (Ishikawa *et al.*, 1985 ▸). Asymmetric polarization-dependent fluctuations could be identified to persist beyond *T*_c_ (Roessli *et al.*, 2002 ▸, 2004 ▸).

In the skyrmion phase, a multitude of non-reciprocal modes are excited for momentum transfers perpendicular to the skyrmion plane. On the other hand, for momentum transfers inside the skyrmion plane, the magnon modes back-fold into the first magnetic Brillouin zone and create complicated Landau levels, similar to the band formation in the helical phase (Janoschek *et al.*, 2010*b* ▸; Weber *et al.*, 2022 ▸). In recent work, Soda *et al.* (2023 ▸) confirmed that the asymmetry of the non-reciprocal modes extends to the microelectronvolt region.

An investigation into the evolution of skyrmion dispersion inside the first magnetic Brillouin zone of the helimagnet Cu_2_OSeO_3_, which shares many properties with MnSi, towards the field-polarized regime was published very recently (Che *et al.*, 2024 ▸). Using Brillouin light scattering, they showed how the individual modes of the skyrmion lattice merge into a single field-aligned ferromagnetic mode. For MnSi we previously investigated the transitions from the conical phase (Weber *et al.*, 2018*b* ▸) and found a qualitatively similar picture for the transition towards the ferromagnetic dispersion.

First studies on the magnetization dynamics at the skyrmion–paramagnetic transition were performed by Schwarze *et al.* (2015 ▸) and Kindervater *et al.* (2019 ▸), where they could discern the changes of the modes from the different phases using microwave techniques. The microwave technique that both groups employed operates at zero momentum transfer, *q* = 0, and is thus restricted to probing the centre of the nuclear Brillouin zone. In this specific regime, they could attribute the fundamental excitations of the skyrmion lattice to counter-clockwise and clockwise rotations as well as a breathing motion of the skyrmions.

For our present work we investigated the spin excitations in MnSi close to the transitions from the skyrmion phase under increasing temperatures. Our goal was to test the stability and evolution of the magnons of the skyrmion phase and find out whether we could identify a clear separation of the excitations across the phases. We employed inelastic neutron scattering, which does not restrict us to zero momentum transfer.

## 2. Skyrmion–paramagnetic transition

### 2.1. Overview and experimental setup

As part of a larger investigation into the stability of the magnon modes at the border of the skyrmion phase, the principal part of the present experiment focused on the skyrmion–paramagnetic transition. The experiment was conducted at the cold-neutron triple-axis spectrometer IN12 (Schmalzl *et al.*, 2016 ▸) at the Institut Laue–Langevin (ILL). We used horizontal collimations of 30′ both before and after the MnSi sample. The instrument possesses a velocity selector in the neutron guide before the monochromator crystals. In addition, a cooled beryllium crystal was placed in the instrument’s *k*_f_ axis between the MnSi sample and the instrument’s analyser crystals. The beryllium crystal suppresses residual higher-energy neutrons not completely removed by the velocity selector, prevents spurious higher-order contamination of the analyser and reduces the instrument’s background. For the experiment, we chose fixed final wavenumbers in the range of *k*_f_ = 1.4–1.5 Å^−1^.

**Figure 1:**
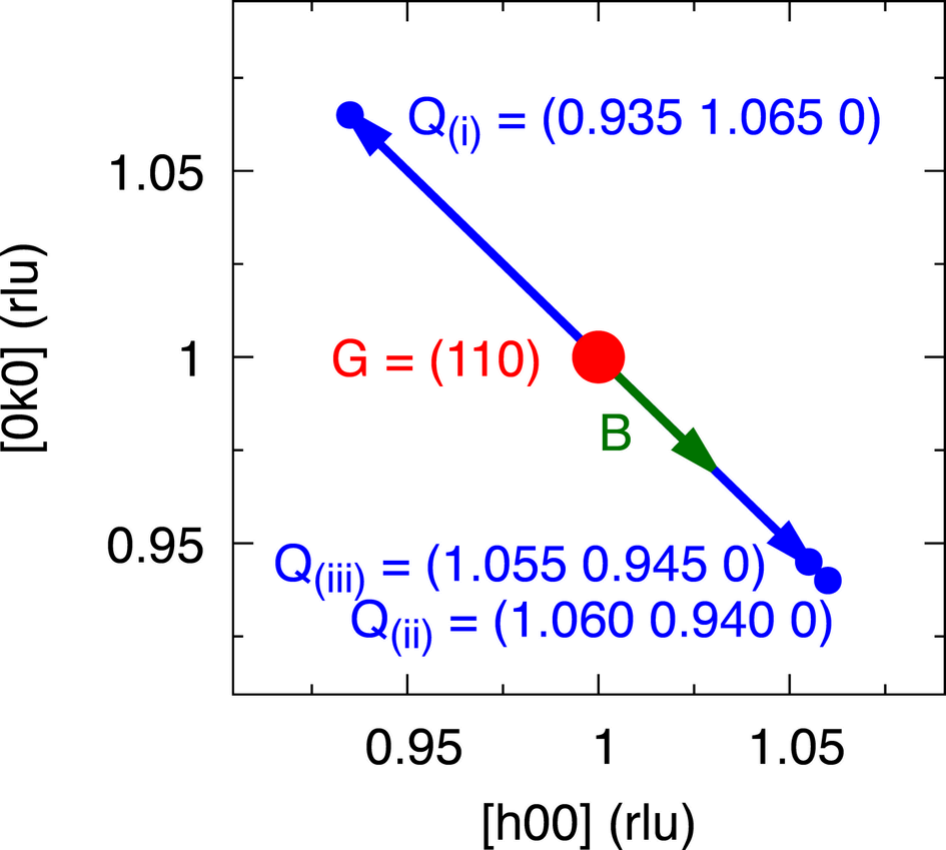
Experimental setup showing the two principal momentum transfers, **Q**_(i)_ and **Q**_(ii)_. We measured in the 〈*hk*0〉 scattering plane at total momentum transfers **Q** = **G** + **q** and external field **B** transverse to the **G** = (110) Bragg peak. Within the instrumental resolution, **Q**_(iii)_ is virtually the same as **Q**_(ii)_.

We used the same single-crystal sample as in our previous studies (Weber *et al.*, 2022 ▸, 2019 ▸, 2018*a* ▸,*b* ▸). The crystal is of cylindrical geometry, with a diameter of 1 cm and a height of 3 cm, and has a mass of ∼15 g, with the cylinder’s long axis pointing along a [001] direction. The sample was mounted in a horizontal Oxford magnet (ILL, 1999 ▸) with the [100] and [010] directions in the horizontal scattering plane. The magnetic field was set to *B* = ±195 mT along 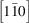. The given value for *B* corresponds to the magnitude of the externally applied field, whereas for the calculations we also take into account the demagnetization factor due to the sample geometry (Sato & Ishii, 1989 ▸). The scattering geometry around the (110) Bragg peak is depicted in Fig. 1 ▸. There, the principal scan positions are marked as **Q**_(i)_ and **Q**_(ii)_, where the total momentum transfer **Q** is defined as the sum of the reciprocal lattice vector and the reduced momentum transfer, **Q** = **G** + **q**. A third scan position, **Q**_(iii)_, principally corresponds to **Q**_(ii)_ within the instrumental resolution.

**Figure 2:**
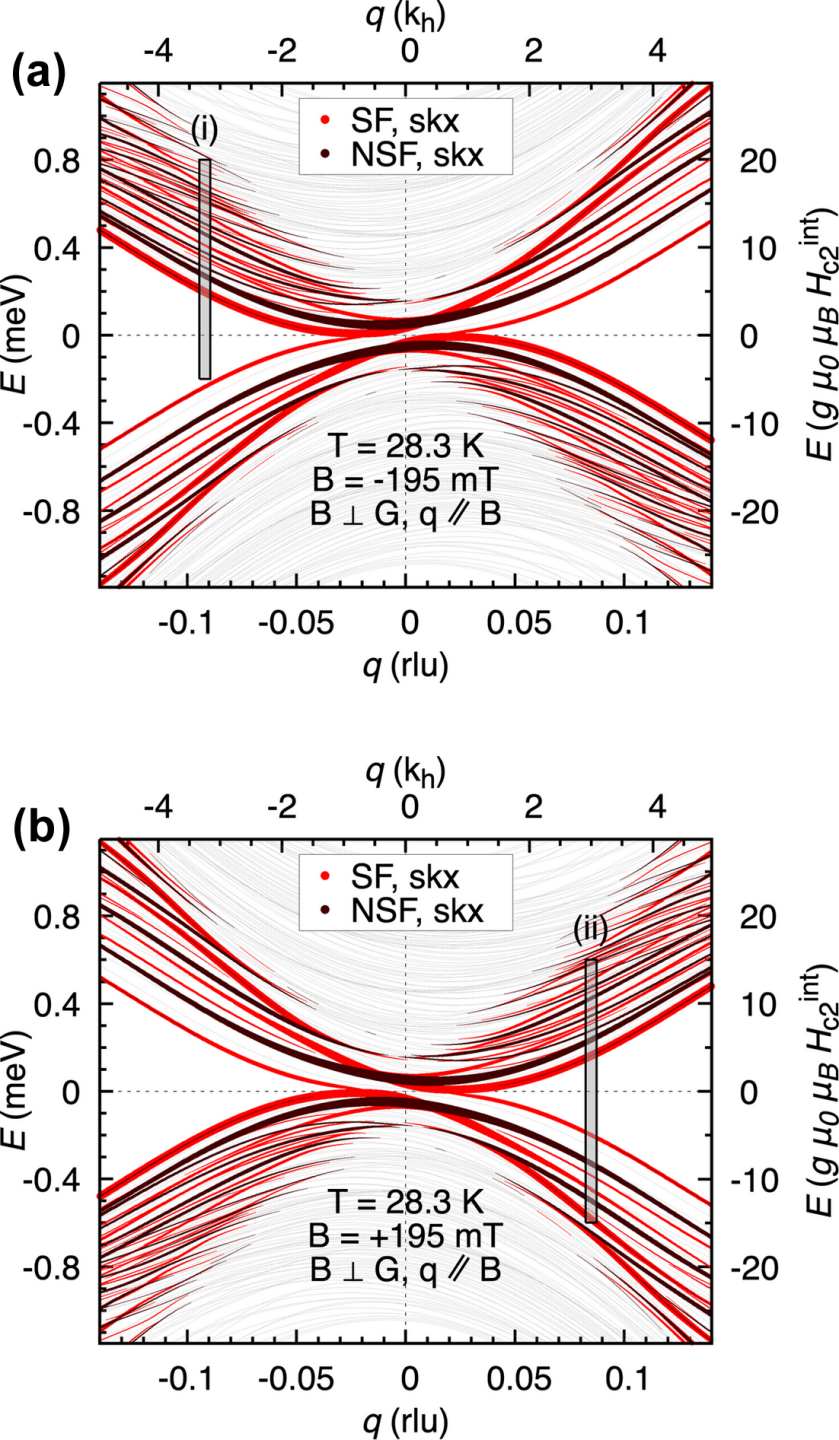
Calculations using our previously developed linear spin-wave model (Garst *et al.*, 2017 ▸; Waizner, 2016 ▸; Weber *et al.*, 2022 ▸) for the skyrmion order at 28.3 K. The plots show the magnon modes propagating along 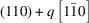. Spin-flip (SF) and non-spin-flip (NSF) components of the scattering cross sections are depicted as red and black curves, respectively. The experiment itself was unpolarized, summing the NSF and SF channels. The thickness of the lines symbolizes the spectral weights of the modes. The grey bars labelled (i) and (ii) mark the positions of the scans in Fig. 3 ▸. Panels (*a*) and (*b*) depict the dispersion for 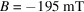 and 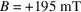, respectively.

Fig. 2 ▸ depicts the results of a theoretical calculation based on our previously developed model (Garst *et al.*, 2017 ▸; Waizner, 2016 ▸; Weber *et al.*, 2022 ▸) of the magnon dispersion in the skyrmion phase. It is dominated by energetically closely spaced parabolic magnon modes. The theoretical model is based on the Landau–Lifshitz equation and takes into account symmetric exchange, Dzyaloshinskii–Moriya and Zeeman terms (Waizner, 2016 ▸), and a higher-order correction in the gradient expansion (Kugler *et al.*, 2015 ▸). The non-reciprocal nature of the excitations is evidenced by the off-centring of the branches. Both panels of Fig. 2 ▸ show the same range of momentum transfer, with only the direction of the field inverted in panel (*b*) with respect to (*a*). The thickness of the lines corresponds to the spin–spin correlation function, which yields the spectral weight of a mode. The abscissas of the plots show the reduced momentum transfer both in reciprocal lattice units, rlu, and in helix wavenumbers, 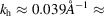

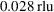
. The ordinates show the energy transfer in millielectronvolts and in units relative to the energy at the conical-field polarized phase transition, 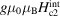, with *g* ≈ 2 for electrons and 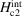 the sample’s internal field at the transition, including demagnetization effects (Sato & Ishii, 1989 ▸). The centre of the figure corresponds to *q* = 0, where the clockwise, counter-clockwise and breathing modes of the skyrmion lattice had been discerned previously (Schwarze *et al.*, 2015 ▸; Kindervater *et al.*, 2019 ▸). At finite momentum transfer, *q* ≠ 0, a multitude of modes appear.

For the experiment we chose momentum-transfer vectors **q**_(i)_ and **q**_(ii)_ where the magnon modes of the skyrmion phase are clearly visible; these are shown as grey vertical bars labelled (i) and (ii) in Fig. 2 ▸. In the present experiment, we repeated the same scans that we performed in the skyrmion phase for increasing temperatures where the skyrmion lattice vanishes and gives way to paramagnetism.

### 2.2. Results

#### 2.2.1. Elastic scattering

**Figure 3:**
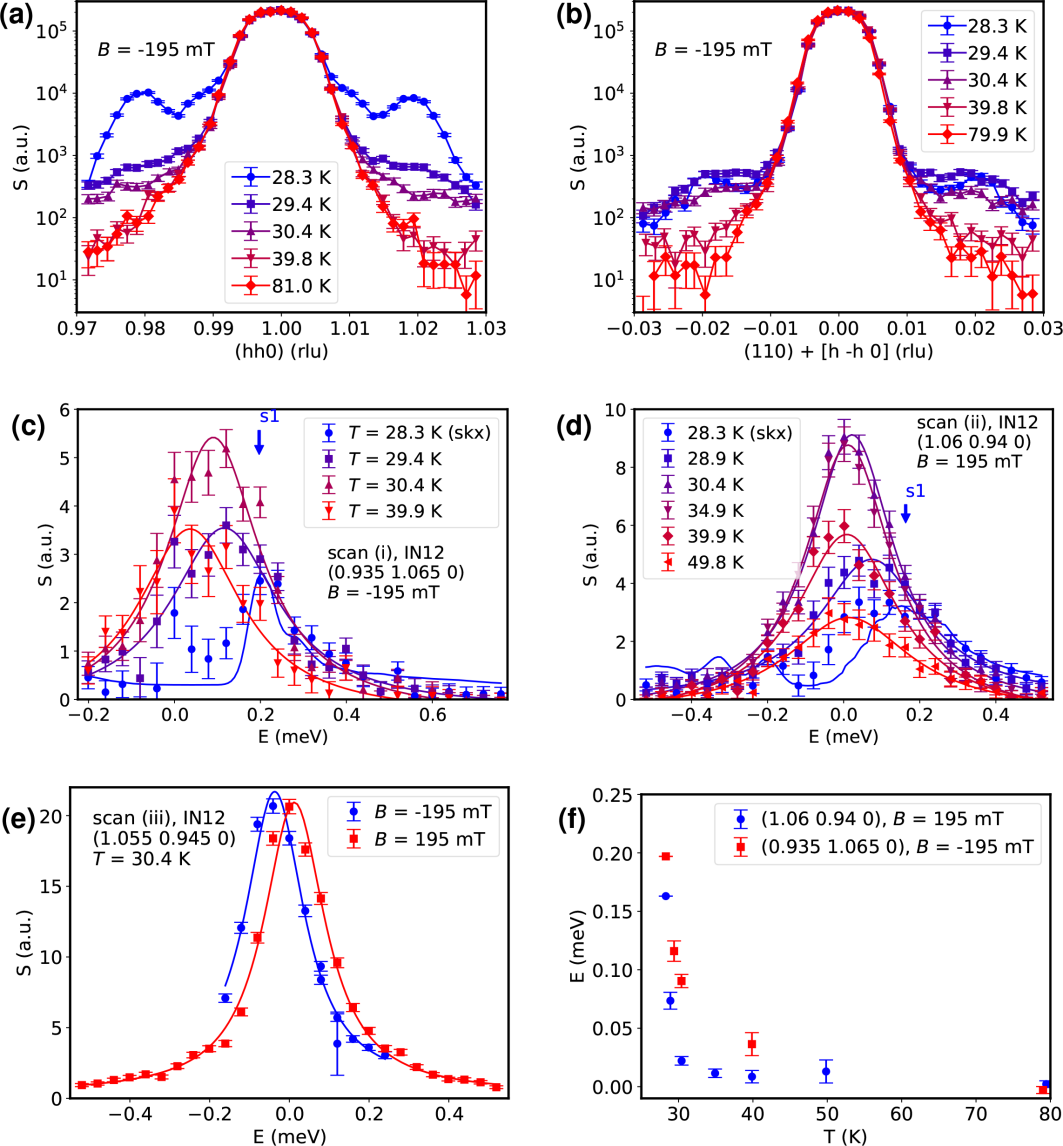
(*a*) Longitudinal elastic scan around (110), where *S* refers to the dynamical structure factor. (*b*) Transvere elastic scan around (110). (*c*), (*d*) Inelastic scans showing the temperature-dependent evolution of the magnon modes starting from the skyrmion phase at *T* = 28.3 K up to the nonmagnetic phase. The scans in panels (*c*) and (*d*) correspond to respective positions (i) and (ii) marked in Fig. 2 ▸. The complicated magnon structure in the skyrmion phase is lost when increasing the temperature. The solid lines for the skyrmion phase at *T*_skx_ = 28.3 K are resolution-convolution simulations of the magnon model (Garst *et al.*, 2017 ▸; Weber *et al.*, 2022 ▸); all the other solid lines are Lorentzian fits. The label s1 marks the position where the first excitation of the skyrmion lattice is expected. (*e*), (*f*) The non-reciprocity that is characteristic of the ordered magnetic phases is retained throughout the paramagnetic phase and only disappears in the clearly non-magnetic regime for *T* > 80 K. It manifests itself via a time-reversal asymmetry that is evident when flipping the polarity of the magnetic field. Panel (*e*) shows the paramagnons for *T* = 30.4 K. Here, the solid lines are Lorentzian fits. In panel (*f*) the asymmetric energy maxima are plotted against temperature.

As a first step we determined the exact temperatures of the phase boundaries. Panels (*a*) and (*b*) of Fig. 3 ▸ show longitudinal and transverse elastic scans, respectively, around the (110) nuclear Bragg peak. Two skyrmion satellite reflections are clearly visible in panel (*a*) at *T* = 28.3 K and *q* = ±0.02; the projections onto the scattering plane of the other four peaks can be discerned at *q* = ±0.01. The projections originate from four skyrmion peaks that are above and below the 〈*hk*0〉 plane, but are observable in plane due to the instrument’s resolution. The peaks disappear towards higher temperatures. Between *T* = 29.4 K and *T* = 30.4 K, the spherically spread elastic signal (Janoschek *et al.*, 2013 ▸) of the fluctuation-disordered phase is visible. For still higher temperatures, all magnetic satellites disappear.

#### 2.2.2. Inelastic scattering

In all of the previously identified phases we performed inelastic neutron scattering as the second step. Fig. 3 ▸(*c*) depicts how the magnon modes of the skyrmion lattice at **Q**_(i)_ = (0.935, 1.065, 0) and *T* = 28.3 K merge into paramagnetic excitations (*T* = 29.4–39.9 K) and finally vanish for high temperatures (*T* = 79 K). Purely non-magnetic data collected at *T* = 79 K were subtracted from all other data sets. The full non-subtracted data sets are shown in Appendix *A*[App appa].

The curves shown for the *T* = 28.3 K data in Fig. 3 ▸(*c*) are Monte Carlo resolution convolutions (Popovici, 1975 ▸; Weber, 2023 ▸) of the instrumental resolution and the theoretical linear spin-wave model (Garst *et al.*, 2017 ▸; Weber *et al.*, 2022 ▸) that we described in the previous section; all other curves are simple Lorentzian fits, which have been found to describe the paramagnons well. The spin-wave model for the skyrmion phase itself is parameter free; the only free variable for the resolution convolution was a global scaling parameter for the dynamical structure factor.

The modes of the skyrmion lattice cannot be discerned individually as they are far below the resolution limit of any triple-axis spectrometer, but they can be well reproduced via the convolution of the theory, where they appear as broad bands in the spectrum. The results show that upon leaving the skyrmion phase by heating the sample the little signatures discernible in the skyrmion phase broaden and merge into featureless nearly quasi-elastic spectra.

In Fig. 3 ▸(*d*) the **Q**_(ii)_ = (1.06, 0.94, 0) magnon modes in the skyrmion phase are visible, but they are not as pronounced as in the previous measurement, because here positive energy transfer corresponds to transverse defocusing of the instrument. This served as a check against possible spurions, namely Bragg tails (Shirane *et al.*, 2002 ▸), in the focusing scans. Bragg tails are remnants of strong Bragg peaks that appear as false inelastic signals due to the correlation of momentum and energy in the instrumental resolution function (Popovici, 1975 ▸). In the skyrmion phase, this problem is especially severe as each of the magnetic satellite reflections generates a Bragg tail in addition to the nuclear peak. The polarity of the magnetic field is flipped for the scans at momentum transfer **Q**_(ii)_ with respect to the setup used for **Q**_(i)_. As we simultaneously invert the direction of the reduced momentum, **q**_(ii)_ = **Q**_(ii)_ − **G**_(110)_, in comparison with **q**_(i)_ = **Q**_(i)_ − **G**_(110)_ the physics does not change. Non-reciprocity implies that the original dispersion and dynamical structure factor is recovered when changing the signs of both field and reduced momentum.

As before, the *T* = 28.3 K curve is a resolution convolution of the model (Garst *et al.*, 2017 ▸; Weber *et al.*, 2022 ▸); all other curves are Lorentzian fits. High-*T* data have been subtracted. As the paramagnons move to lower energies for increasing temperatures, their intensities increase due to the Bose factor. Their intensities observed around *E* = 0 reach a maximum at approximately *T* = 35 K; for even higher temperatures they decrease again until only nuclear-incoherent scattering is left at *T* > 79 K.

The non-reciprocal character of the magnetic excitations, which is found in all of the ordered phases, is retained in the paramagnetic regime but becomes less pronounced as the temperatures approach the non-magnetic phase. Fig. 3 ▸(*e*) shows a scan at **Q**_(iii)_ = (1.055, 0.945, 0) in the paramagnetic regime at 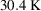 for two directions of the external field. This visualizes the time-reversal asymmetry that is observed upon inverting the direction of the magnetic field. The same dispersion would only be recovered when flipping both the reduced momentum transfer and the magnetic field direction at the same time. Within the instrumental resolution the points **Q**_(iii)_ and **Q**_(ii)_ and their dispersions are virtually the same (see Fig. 1 ▸); we only distinguish between them for technical reasons.

Due to non-reciprocity, the spin excitations are not centred around *E* = 0, even though the system is deep in its paramagnetic state. The temperature-dependent shifts of the maxima of the scattering intensity are plotted in Fig. 3 ▸(*f*) for the two principal scan positions, **Q**_(i)_ and **Q**_(ii)_. For the data outside the skyrmion phase, the energy shifts were obtained from the centres of the Lorentzian fits shown in panels (*c*) and (*d*). For the data measured inside the skyrmion phase, we calculated the energies of the modes using our theoretical model. At temperatures as high as 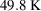 an energy offset can still be discerned.

## 3. Helimagnetic–paramagnetic transition

The final part of the experiment concerned the helimagnetic–paramagnetic transition for no applied external field. A large MnSi single crystal of ∼50 g was oriented in an 〈*hk*0〉 scattering plane and placed in a standard ILL Orange cryostat. As in the first part, the experiment was conducted using horizontal collimators of 30′ both before and after the MnSi sample and with a beryllium crystal in the instrument’s *k*_f_ axis. We used a fixed *k*_f_ of 1.5 Å^−1^.

**Figure 4:**
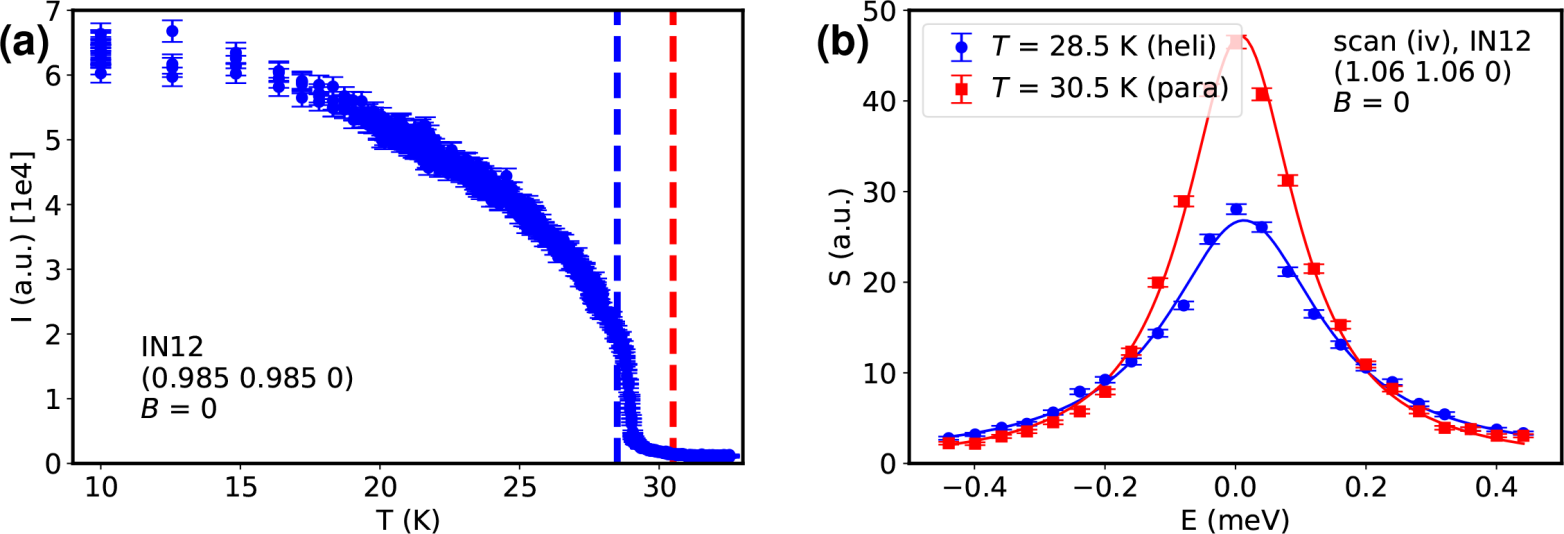
(*a*) Elastic scan of one of the helimagnetic satellites versus temperature. The phase transition towards paramagnetism is observed at *T*_c_ = 29 K. The vertical dashed lines mark the scan temperatures. (*b*) Magnon modes in the multi-domain helical (*T* = 28.5 K) and the paramagnetic (*T* = 30.5 K) phase at **Q** = (1.06, 1.06, 0) close to their phase boundary. The solid lines are Lorentzian fits.

As no magnetic field was applied, the helimagnetic state comprises four domains with the helices aligned along the 〈111〉 directions. Magnons emanating from all four domains overlap in this state (Janoschek *et al.*, 2010*a* ▸). Fig. 4 ▸(*a*) tracks the intensity of a projection onto the [110] direction of one of these peaks against temperature. The phase transition towards paramagnetism sets in at *T*_c_ = 29 K.

Fig. 4 ▸(*b*) depicts the spin excitations of both the helimagnetic and the paramagnetic state at **Q** = (1.06, 1.06, 0) close to their common phase boundary. The overlap of magnons from four magnetic domains is shown by the broad tails around the central incoherent peak (Γ_FWHM_ = 0.287 ± 0.006 meV). Heating the sample beyond the phase transition shifts the peak of the intensity towards lower energies, which manifests itself by a higher amplitude but lower width of the observed peak (Γ_FWHM_ = 0.207 ± 0.003 meV). The values are from Lorentzian curve fits; a resolution deconvolution or convolution simulation was not attempted. At zero field, the line shapes are entirely symmetrical; a non-reciprocity is not observed.

## 4. Summary and discussion

We investigated the transition of the magnon dispersion from the skyrmion to the paramagnetic phase of MnSi at momentum transfers that are not restricted to the Brillouin zone centre.

Measuring the excitations of the skyrmion phase and beyond proves challenging, since the energy scales are on the brink of what is resolvable with inelastic neutron scattering. In the skyrmion phase, the magnons comprise a plethora of individual modes, which, while not being observable individually, can be well reproduced by convoluting the linear spin-wave model with the instrumental resolution.

While the data alone suggest a smooth transition in temperature upon changing into the paramagnetic state, we infer from the convolution of the theoretical model that the magnons still experience a clear transition as far as the internal details of the dispersion are concerned. Above the transition temperature, the complicated inelastic modes give way to quasi-elastic broadenings while retaining their non-reciprocal characteristics. In the fluctuation-disordered phase, a strong increase in intensity can be observed, which is due to the Bose occupation factor at very low energies. In the paramagnetic state at high temperature, the intensity of the signal gradually decreases again for increasing temperatures.

The seemingly smooth transition despite the first-order nature of the skyrmion–paramagnetic phase boundary (Bauer *et al.*, 2013 ▸) could be taken as an indication of a mixed phase. Such a mixed phase could be understood as either paramagnetic fluctuations being present in the skyrmion phase or characteristics of the skyrmions surviving into the paramag­netic state. As the elastic scans show that the paramagnetic state is clearly separated from the skyrmion phase by the onset of the smeared-out fluctuations of the fluctuation-disordered paramagnetic phase, which cannot be observed in the skyrmion order, we rule out the first case. The second case would be in line with a previous study that suggests that skyrmion correlations exist in the fluctuation-disordered phase well beyond the transition temperature (Kindervater *et al.*, 2019 ▸).

The second result of our study shows that the non-reciprocity of the magnons in the skyrmion phase is retained even for high temperatures inside the paramagnetic regime. This result is not surprising due to the applied magnetic field. The asymmetric energy shift diminishes with increasing temperature. A similar result was also obtained for the very low energy excitations in the microelectronvolt region that were measured by Soda *et al.* (2023 ▸). They put forth the hypothesis that the paramagnetic fluctuations remain in a skyrmion-like state (Soda *et al.*, 2023 ▸). We believe in a simpler interpretation, namely that the presence of an external field causes strong fluctuations in the paramagnetic phase where long-range collective modes are still partially possible. Apart from the external field, the persisting non-reciprocal character stems from the Dzyaloshinskii–Moriya interaction, which originates from the crystal’s symmetry alone.

No non-reciprocal characteristics were observed for the helimagnetic–paramagnetic transition at zero field, where time-reversal symmetry is not broken. Even though they were not detectable in the present experiment, minute polarization-dependent asymmetries in the chiral fluctuations still persist into the paramagnetic phase and could still be observed by Roessli *et al.* (2002 ▸) using linear polarization analysis.

Materials with persisting non-reciprocal responses well above the critical temperature may prove especially interesting for research in the field of magnonics. A magnonic component such as a unidirectional field guide would not need to be cooled to the onset of ordered magnetism but could function at higher temperatures.

This work sets the stage for future investigations into the dynamics of the magnons at the border of the conical and the skyrmion phase where we expect to observe similar transitional effects.

## Figures and Tables

**Figure 5:**
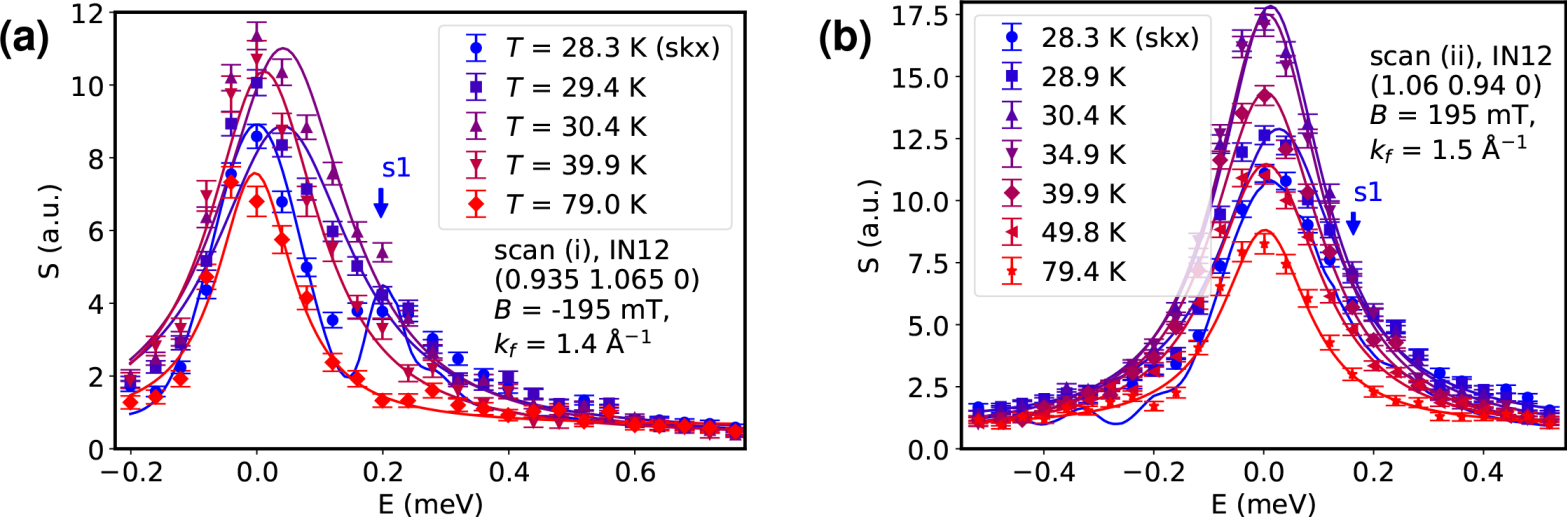
Unsubtracted data sets with panels (*a*) and (*b*) corresponding to Figs. 3 ▸(*c*) and 3 ▸(*d*), respectively. Here, we also show the high-temperature non-magnetic reference scan at *T* = 79 K explicitly. The solid lines for the skyrmion phase at *T*_skx_ = 28.3 K are resolution-convolution simulations of the magnon model (Garst *et al.*, 2017 ▸; Weber *et al.*, 2022 ▸) plus Gaussian profiles modelling the incoherent-elastic contribution; all the other solid lines are Lorentzian fits.

## Data Availability

The data files and the data-analysis scripts are available at https://doi.ill.fr/10.5291/ILL-DATA.CRG-3132. The source code for the model (Weber *et al.*, 2022 ▸) is available at https://zenodo.org/records/17338265.

## Appendix A Unsubtracted data

The raw unsubtracted inelastic data sets for the skyrmion–paramagnetic transition are shown in Fig. 5 ▸.
